# Neovascularization of saphenous veins

**DOI:** 10.1590/1677-5449.190030

**Published:** 2019-05-07

**Authors:** Wolfgang Mouton, Dominik Heim, Jan Janzen

**Affiliations:** 1 Beau-Site Hospital, Bern, Switzerland.; 2 Hohmad Clinic, Thun, Switzerland.; 3 VascPath, Bern, Switzerland.

Neovascularization has been defined as the presence of reflux in a previously ligated sapheno-femoral or sapheno-poplital junction caused by the development of incompetent tortuous veins linked to the thigh or calf varicosities.^1^

Langenbeck already described in 1861 what we would now define as neovascularization^2^: “In one case of very large varix of the great saphena in a young man I had extirpated the enlarged vein in the length of three inches and ligated the upper and lower ends. One year later I found, in the region of the scar tissue of the extirpation, a new vein channel of the thickness of the quill of a crow’s feather, which again joined the both ends of the fully functioning saphena.”^2^

The term neovacularization was first determined in 1987 by Glass as “recurrence through growth of new vessels”.^3^ The title of the MD thesis of Glass was: “Regeneration of veins with particular reference to recurrence of varicose veins of the lower extremity.” Glass also proved neovascularization by histology.^4^ In this experimental study he dissected the great saphenous vein under local anaesthesia in patients with a venous ulcer, then waited until the venous ulcer was healed in order to then perform sapheno-femoral ligation with stripping of the great saphenous vein. The previously transected ends with their surrounding tissue he resected and investigated these specimens by injection of normal physiological solution and subsequentely examined the specimens by radiography and by histopathology.

Histopathology showed that after 6 weeks new venous vessels and after 18 weeks parallel new venous vessels were present and after 40 weeks there was a continuity of venous flow.

With this he could demonstrate by histopathology that very thin-walled irregular new venous vessels did reconnect the previously transected ends of the great saphenous vein.^4^

Neovascularization may represent a physiological healing process following venous surgery. Venous disconnection and altered venous hemodynamics may initiate neovascularization.^1^^,^ 5 Growth factors, matrix metalloproteinases and angiopoietin are supposed to be involved.^1^

**Figure 1:**
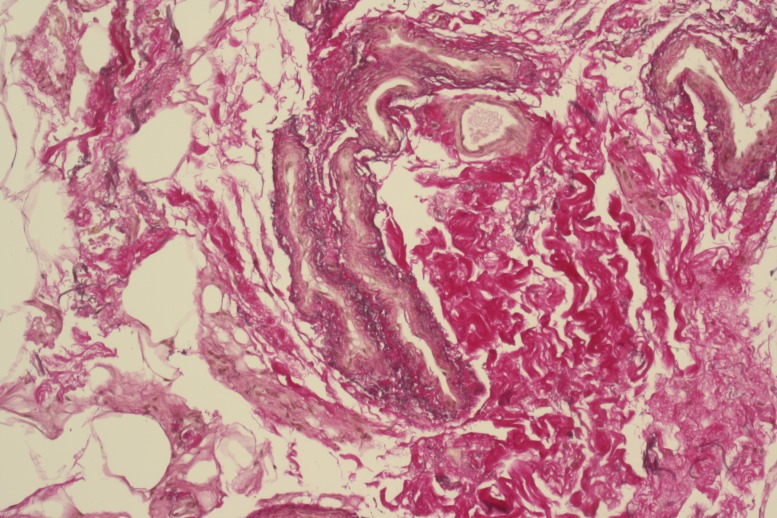
Typical microscopical aspects of neovascularization.

Histological examination of the operative specimen helps to distinguish between different morphological subtypes of neovascularization and “normal” remnants of venous vessels. Morphologically, various patterns exist such as typical neovascularization (Figure 1), venous vessels with leiomyomatous hyperplasia and mixed forms.

Neovascularization is detected in duplex ultrasound as tortuous vessels leading to the stump or connecting end of the great or small saphenous vein. Neovascularization detected by duplex may or may not result in an intervention depending, amongst other factors, on the clinical presentation of the recurrent varicose veins of the patient. Other common causes for recurrent varicose veins apart from neovascularization are tactical error (persistent venous reflux in a saphenous vein secondary to inadequate preoperative evaluation and inappropriate surgery), technical error (persistent venous reflux due to inadequate or incomplete surgical technique) and of course disease progression (development of venous reflux secondary to the natural evolution of the disease).^1^

It is recommended to treat symptomatic recurrent varicose veins if indicated, by endovenous thermal ablation, ultrasound guided foam sclerotherapy or phlebectomies.^1^ Re-exploration of the groin or popliteal fossa is not recommended as the first choice.^1^
